# O.1.2-6 Music and Movement for Health: an arts-based Intervention to promote health and wellbeing of community dwelling older adults in Ireland

**DOI:** 10.1093/eurpub/ckad133.093

**Published:** 2023-09-11

**Authors:** Amanda Clifford, Orfhlaith NiBhriain, Steven Byrne, Pui Sze Cheung, Quinette Louw, Liam Glynn, Hilary Moss, Desmond O'Neill, Catherine B Woods, Ali Sheikhi, Rosemary Joan Gowran, Catherine Maher, Brendan Kennelly, Jon Salsberg, Lehana Thabane

**Affiliations:** School of Allied Health, Ageing Research Centre, Health Research Institute, University of Limerick, Limerick, Ireland; Irish World Academy of Music and Dance, University of Limerick, Limerick, Ireland; School of Allied Health, Ageing Research Centre, Health Research Institute, University of Limerick, Limerick, Ireland; School of Allied Health, Ageing Research Centre, Health Research Institute, University of Limerick, Limerick, Ireland; Irish World Academy of Music and Dance, University of Limerick, Limerick, Ireland; Division of Physiotherapy, Department of Health and Rehabilitation Sciences, Stellenbosch University, Cape Town, South Africa; School of Medicine, Health Research Institute, University of Limerick, Limerick, Ireland; Irish World Academy of Music and Dance, University of Limerick, Limerick, Ireland; Health Research Institute, University of Limerick, Limerick, Ireland; Centre for Ageing, Neuroscience and the Humanities, Trinity College Dublin, Dublin, Ireland; Physical Activity for Health Research Cluster, Health Research Institute, Department of Physical Education and Sport Sciences, University of Limerick, Limerick, Ireland; Health Research Institute, University of Limerick, Limerick, Ireland; School of Allied Health, Ageing Research Centre, Health Research Institute, University of Limerick, Limerick, Ireland; Assisting Living and Learning (ALL) Institute, Maynooth University, Maynooth, Ireland; Rehabilitation Unit, Community Hospital of the Assumption, Thurles, Tipperary, Ireland; Cairnes School of Business and Economics, National University of Ireland Galway, Galway, Ireland; School of Medicine, Health Research Institute, University of Limerick, Limerick, Ireland; Department of Health Research Methods, Institute for Research in Ageing, McMaster University, Canada; Amanda.Clifford@ul.ie

## Abstract

**Purpose:**

The Music and Movement for Health study is a pragmatic cluster-randomised controlled feasibility trial that aims to assess the feasibility of an innovative music and dance intervention that was developed to promote health and wellbeing of community dwelling older adults in Ireland. This study also aims to obtain preliminary data on the effect of the arts-based intervention on health and wellbeing in older adults.

**Methods:**

One hundred community-dwelling adults aged 65 years or older were recruited across seven clusters in the Mid-West region of Ireland. The seven clusters were randomly allocated to either the intervention group (n = 56, 4 clusters) or the control group (n = 44, 3 clusters). The intervention group participated in the Music and Movement for Health intervention which consisted of a 1.5-hour session and a 1-hour home-based programme weekly for 12-weeks. Music and Movement for Health is a complex intervention developed following the Medical Research Council Framework, co-designed with stakeholders using research evidence and theories to optimise engagement. Participants completed assessments at baseline and after 12 weeks and participated in focus group or interview after their participation.

**Results:**

The targeted feasibility outcomes including the recruitment rates, the retention rates, attendance, and participation rates were achieved. Randomisation was completed according to the protocol and no safety issues were identified. The outcome measures used were acceptable and feasible for this population. The results on secondary outcomes and process evaluation of this trial are awaited.

**Conclusion:**

This research strives to strengthen collaborative efforts to implement effective, sustainable and cost-effective programmes for older adults to support community connection, enhancing health and wellbeing, in turn reducing demands on the healthcare system. To our knowledge, Music and Movement for Health is the first evidence-based arts intervention that has incorporated music therapy with dance to improve health and wellbeing of community dwelling older adults. Feasibility results demonstrate that Music and Movement for Health is feasible, acceptable and safe for community dwelling older adults in the Mid-West region of Ireland.

